# Effects of Amino Acid Decarboxylase Genes and pH on the Amine Formation of Enteric Bacteria From Chinese Traditional Fermented Fish (Suan Yu)

**DOI:** 10.3389/fmicb.2020.01130

**Published:** 2020-07-02

**Authors:** Qin Yang, Ju Meng, Wei Zhang, Lu Liu, Laping He, Li Deng, Xuefeng Zeng, Chun Ye

**Affiliations:** ^1^School of Liquor and Food Engineering, Guizhou University, Guiyang, China; ^2^Guizhou Provincial Key Laboratory of Agricultural and Animal Products Storage and Processing, Guiyang, China; ^3^Key Laboratory of Animal Genetics, Breeding and Reproduction in the Plateau Mountainous Region, Ministry of Education, Guiyang, China; ^4^College of Food Science and Engineering, Wuhan Polytechnic University, Wuhan, China

**Keywords:** biogenic amines (BAs), enteric bacteria, amino acid decarboxylase genes, pH, Suan yu

## Abstract

The formation of biogenic amines (BAs) is an important potential risk in Suan yu. This study investigated the amine production abilities of 97 strains of enteric bacteria screened from Suan yu. The genotypic diversity of amino acid decarboxylase and the effect of pH were explored on 27 strains of high-yield BAs. Results showed that high levels of putrescine, histamine, and cadaverine were produced by the 97 strains. In addition, 27 strains carried *odc*, *speA*, *speB*, *adiA*, and *ldc* genes. Thirteen carried *hdc* gene. *Morganella morganii* 42C2 produced the highest putrescine content of 880 mg/L via the ornithine decarboxylase pathway. The highest histamine content was produced by *Klebsiella aerogenes* 13C2 (1,869 mg/L). The highest cadaverine content was shown by *Klebsiella pneumoniae* 47C2 (1,821 mg/L). *odc*, *adiA*, *speB*, *ldc*, and *hdc* play important roles in the cellular acid stress response. Acid stress caused the growth delay but can increase the contents of putrescine, histamine, and cadaverine. Decarboxylase was strain specific rather than species specific. This study provides a reference for the genotypic diversity of decarboxylase and effect of pH on the types and abilities of BAs produced by enteric bacteria in Suan yu.

## Introduction

Suan yu is a Chinese traditional fermented whole fish snack with a characteristic flavor in ethnic minority areas (Miao and Dong minorities) in Southwest China. Different from other fermented fish products, Suan yu is free of fishy odor and taste, features strong fermentation flavor, retains all the nutritional advantages of fish, and remains stable during storage (Zeng et al., 2013b; Gassem, 2017). However, Suan yu is obtained in traditional workshops. The process of Suan yu making provides an ideal condition for the formation of BAs. On the one hand, the raw material processing is random, unhygienic practices, and substandard manufacturing practice can cause growth of spoilage microorganisms (Gardini et al., 2016; Doeun et al., 2017). On the other hand, during natural fermentation, fermented fish gradually acidifies, protein is degraded, and free amino could be transformed into BAs by microbial decarboxylases (Zeng et al., 2013a; Ishimaru et al., 2019; Xu et al., 2019). Once produced, BAs become difficult to eliminate by high-temperature treatment, refrigeration, cooking, or technological treatment (Lu et al., 2015; Ozogul et al., 2015; Tofalo et al., 2016). If ingested in large amounts, BA can trigger human health problems leading to hypertension, palpitations, vomiting, flushing, and headaches (Bover-Cid et al., 2014).

Zeng et al. (2013b) reported the chemical and microbial properties of six brands of Suan yu from different locations in China. *Enterobacteria* were detected, and putrescine, cadaverine, and histamine formed in the six brands. The pH values of Suan yu range from 4.27 to 5.18. A large number of researchers have reported the formation and accumulation of BAs in fish and fish products due to the presence of decarboxylase-producing microorganisms, especially enteric bacteria (Torido et al., 2014). *Morganella morganii*, *Klebsiella pneumoniae*, and *Enterobacter aerogenes* strains of enteric bacteria from fresh fish show decarboxylase activity and decarboxylates amino acid to the biogenic amine (BA) (Torido et al., 2012; Bjornsdottir-Butler et al., 2010). Some enteric bacteria (*E. cloacae*, *Citrobacter freundii*) from fish paste and dried milkfish produce histamine (Hsu et al., 2009; Naila et al., 2011). *Escherichia coli*, *Klebsiella pneumoniae*, and *Klebsiella oxytoca* produced putrescine (Wunderlichová et al., 2013).

Decarboxylation of free amino acids by microorganism decarboxylases produces BAs (Latorremoratalla et al., 2014). The production of decarboxylases is highly correlated with the presence of decarboxylase genes. In enteric bacteria, substantial amounts of putrescine can be formed in two metabolic pathways. In the first pathway, putrescine can be synthesized directly from the decarboxylation of ornithine by ornithine decarboxylase enzyme (*odc*). In the second pathway, it is produced indirectly from the decarboxylation of arginine by arginine decarboxylase (*speA*, *adiA*) via agmatine. Agmatine is subsequently converted to putrescine by agmatinase (*speB*) (Wunderlichová et al., 2015), cadaverine via lysine decarboxylase (*ldc*), and histamine via histidine decarboxylase (*hdc*) (de las Rivas et al., 2006; Bjornsdottir et al., 2009; Wunderlichová et al., 2013).

Environmental factors (i.e., free amino acid, pH, temperature, oxygen supply, NaCl) influence the ability of BA-producing species/strains and bacterial growth (Takahashi et al., 2015; Rio et al., 2016). pH is an important environmental factor that influences bacterial growth and amino acid decarboxylase formation and activity (Usheer et al., 2011; Chiara et al., 2014). BAs form in fermented fish during the ripening step, in which a large number of proteins are degraded to form free amino acids, and the pH decreases gradually. Under the cellular acid stress response, abundant free amino acids could be transformed into BAs by microbial decarboxylases to withstand acidic environments, and decarboxylation increases survival (Álvarez-Ordóñez et al., 2010; Usheer et al., 2011; Chiara et al., 2014). qRT-PCR experiments showed that the *hdc* and *hisRS* genes are highly induced under acidic and histidine-rich conditions and that the optimum pH for the hdc activity of enteric bacteria ranges from 5.0 to 6.5 (Chiara et al., 2014; Bjornsdottir-Butler et al., 2015). The *ldc* expression of *E. coli* is induced at acidic pH and at high concentrations of the respective amino acids (Park et al., 2017). In addition, previous reports indicated that the polyamines formed by the arginine decarboxylase biosynthetic *speA* (constitutive decarboxylases) are significant cellular components and are involved in some processes, including DNA replication, transcription, membrane permeability, protein synthesis, and biofilm formation. Meanwhile, the arginine decarboxylase biodegradative *adiA* (inducible decarboxylases) is mainly involved in cellular acid stress response and the presence of high-concentration arginine (Álvarez-Ordóñez et al., 2010; Kanjee et al., 2011).

However, limited information is available about the production of BAs by the high production of BAs in enteric bacteria isolated from Suan yu. Therefore, the present work aimed to assess BA production by strains of enteric bacteria from different fermentation stages of Suan yu, detect the genotypic diversity of amino acid decarboxylase by PCR, conduct a phenotypic analysis of the BA production by enteric bacteria, and determine the effect of acid stress on bacterial amine-production ability by RP-HPLC (Coton et al., 2010; Landete et al., 2011; Wunderlichová et al., 2015). This study can be used as a reference for the genotypic diversity of decarboxylase of BA-producing bacteria and effect of pH on the types and abilities of BAs produced by enteric bacteria in protein-rich fermented foods.

## Materials and Methods

### Suan Yu Preparation, Strain Isolation, and Growth Conditions

Fresh carps (*Cyprinus carpio* L.) were purchased from a local market (Guiyang, Guizhou, China). Suan yu samples were prepared in accordance with the method described by Zeng et al. (2013b). The samples were withdrawn and analyzed on days 3, 7, 12, 18, and 25. Each sample (25 g) was placed in a sterile plastic bag and homogenized with 225 ml of 0.85% NaCl. The homogenized samples were diluted and then placed in violet red bile glucose agar for enterobacteria growth at 37°C for 1–2 days.

### Reference Bacterial Strain

Biogenic amine-producing bacterial strain was purchased from Shanghai Biology Collection Center (Shanghai, China): the histamine-producing *Photobacterium phosphoreum* CECT 4192, the cadaverine-producing *S. sonnei* CECT 457 (ATCC 11060), the putrescine-producing *Escherichia coli* K-12 substr. MG1655 (NC_000913) (de las Rivas et al., 2005, 2006; Wunderlichová et al., 2013).

### Detection of Amine-Forming Ability

Production of BAs was tested by inoculating isolates (7–9 log CFU ml^–1^) directly into tubes containing 10 ml of mixed amino acid decarboxylase media. The medium described by Møller (1954) was used to culture the enteric bacteria. The media were supplemented with L-histidine, L-lysine, L-arginine, and L-tyrosine at 0.5% final concentration and L-ornithine monohydrochloride (Solarbio, China) at 0.25% final concentration. Pyridoxal-5-phosphate (Macklin, China) was included in the media (at 0.005%).

### Biogenic Amine Analysis From Bacterial Cultures by RP-HPLC

Biogenic amines were extracted from the samples in accordance with the procedures developed by Ben-Gigirey et al. (1998) and Curiel et al. (2011) with a slight modification. Exactly 2 ml of the broth media was centrifuged at 12,000 × *g* for 10 min, and then 1 ml of the supernatant was extracted with 1 ml of 0.4 M perchloric acid for 30 min. BA derivatization was carried out in accordance with the procedures developed by Ben-Gigirey et al. (1998), Zhai et al. (2012), and Zeng et al. (2013b) with a slight modification. A 1-ml aliquot of the extract or a standard solution was mixed with 200 μl of 2 M sodium hydroxide and 300 μl of saturated sodium bicarbonate. Under alkaline conditions, 2 ml of dansyl chloride (10 mg/ml) was mixed with each sample and then incubated for 45 min at 40°C. Residual dansyl chloride was removed by adding 100 μl of 25% ammonium hydroxide. The mixture was incubated for 30 min at room temperature and complemented to 5 ml with acetonitrile. Finally, the supernatant was filtered through 0.2-mm-pore-size filters prior to RP-HPLC analysis (Agilent l260; United States). Liquid chromatographic separations were performed using Zorbax SB-C18 (4.6 × 250 mm). Mobile phases consisted of acetonitrile (A) and water (B). The column temperature and flow rate were set at 35°C and 1 ml min^–1^, respectively. The UV detection wave length was 254 nm. The gradient program is shown in Table 1.

**TABLE 1:** RP-HPLC elution program for the BAs analysis.

**Time (min)**	**Acetonitrile (%)**	**H_2_O (%)**
0	60	40
15	75	25
22	85	15
25	90	10
28	90	10
35	60	40

### DNA Extraction and Strain Identification

Total DNA was extracted from bacterial cultures grown to stationary phase using 1 ml of the cultures with the TIANamp Bacteria DNA Kit (TianGen, China) in accordance with the manufacturer’s instructions and stored frozen at −20°C until further analysis.

The 16S rDNA gene PCR amplifications of extracted DNA were performed with the following primers: 27F (5′-AGAGTTTGATCCTGGCTCAG-3′); 1492R (5′-GGTT ACCTTGTTACGACTT-3′). PCR was performed in a 25-μl amplification reaction mixture containing 2.5 μl of template DNA, 12.5 μl of TaqpMIX (Sangon Biotech, China), and 0.4 μM of each primer. The amplification program was as follows: 5 min for enzyme activation at 94°C; followed by 35 cycles of 1 min at 94°C, 1 min at 55°C, and 2 min at 72°C; and a final extension step of 10 min at 72°C (Chen et al., 2010). BLAST analysis was conducted to confirm the bacterial species.

### Detection of Amino Acid Decarboxylase Genes in the Biogenic Amine-Producer Strains

DNAs from 27 strains were subjected to PCR amplification to detect the presence of amino acid decarboxylase genes. The primer pairs 106/107, PUT1-F/PUT1-R, and CAD1-F/CAD1-R were used to detect the *hdc*, *odc*, and *ldc* gene fragments, respectively (de las Rivas et al., 2005, 2006). The specific primers adc5F/adc5R, adiA3F/adiA3R, and agm4F/agm4R were used to detect the *speA*, *adiA*, and *speB* gene fragments, respectively (Wunderlichová et al., 2013). The primer sequences are listed in Table 2. All primers were synthesized by Sangon Biotech (China). PCR reaction was performed in a 20-μl amplification reaction mixture containing 2 μl of template DNA, 10 μM of each primer, 20 mM Tris-HCl (pH 8.0), 50 mM KCl, 2.5 mM MgCl_2_, 200 μM of each deoxynucleoside triphosphate, and 0.5 U of DNA polymerase (Solarbio, China). The amplification programs are listed in Table 3. The PCR products were separated on a 2% agarose gel in 1 × TAE (Tris-acetate/EDTA), stained with GenGreen, and then visualized under a UV lamp (Universal Hood II, Biorad).

**TABLE 2:** Primers used for the PCR targeting BA-production associated genes.

**Primer**	**Sequence (5′ to 3′)**	**Product (bp)**	**Target element**	**References**
adc5F/adc5R	CAAYTTCTCGSTGTTCCAG	282	Arginine decarboxylase-biosynthetic (*speA*) gene	Wunderlichová et al. (2013)
	TCRCCRAACAGGTTGTGC			
adiA3F/adiA3R	TKCCAASCCGYAACCG	548	Arginine decarboxylase-biodegradative (*adiA*) gene	Wunderlichová et al. (2013)
	AACMGCTTCRTCRATCAC			
agm4F/agm4R	TGAACGTCGTGGACTGCGG	355	Agmatinase (*speB*) gene	Wunderlichová et al. (2013)
	GCRTCCAGCACGGTAAAGC			
PUT1-F/PUT1-R	TWYMAYGCNGAYAARACNTAYYYTGT	1440	Ornithine decarboxylase (*odc*) gene	de las Rivas et al. (2006)
	ACRCANAGNACNCCNGGNGGRTANGG			
CAD1-F/CAD-R	TTYGAYWCNGCNTGGGTNCCNTAYAC	1098	Lysine decarboxylase (*ldc*) gene	de las Rivas et al. (2006)
	CCRTGDATRTCNGTYTCRAANCCNGG			
106/107	AAYTCNTTYGAYTTYGARAARGARG	534	Histamine decaboxylase (*hdc*) gene	de las Rivas et al. (2005)
	ATNGGNGANCCDATCATYTTRTGNCC			

**TABLE 3:** The amplification program using the primer pairs described in Table 2.

**Primer**	**Amplification program**	**References**
adc5F/adc5R	94°C 5 min; 2 cycles of 94°C 45 s, 62°C 1 min, 72°C 1 min; followed by 20 cycles, when annealing temperature was decreased by 0.5°C in every other cycle: 94°C 45 s, annealing from 61 to 52°C 1 min, 72°C 1 min; 10 cycles of 94°C 45 s, 52°C 1 min, 72°C 1 min with a final extension at 72°C for 10 min.	Wunderlichová et al. (2013)
agm4F/agm4R	94°C 5 min; 2 cycles of 94°C 45 s, 62°C 1 min, 72°C 1 min; followed by 20 cycles, when annealing temperature was decreased by 0.5°C in every other cycle: 94°C 45 s, annealing from 61 to 52°C 1 min, 72°C 1 min; 10 cycles of 94°C 45 s, 52°C 1 min, 72°C 1 min with a final extension at 72°C for 10 min.	Wunderlichová et al. (2013)
adiA3F/adiA3R	94°C 5 min; 2 cycles of 94°C 45 s, 62°C 1 min, 72°C 1 min; followed by 30 cycles, when annealing temperature was decreased by 0.5°C in every other cycle: 94°C 45 s, annealing from 61 to 47°C 1 min, 72°C 1 min; 3 cycles of 94°C 45 s, 47°C 1 min, 72°C 1 min with a final extension at 72°C for 10 min.	Wunderlichová et al. (2013)
PUT1-F/PUT1-R	95°C 5 min, 25 cycles of 95°C 45 s, 55°C 1 min, 72°C 2 min with a final extension at 72°C for 10 min.	de las Rivas et al. (2006)
CAD1-F/CAD-R	95°C 5 min, 25 cycles of 95°C 45 s, 55°C 1 min, 72°C 2 min with a final extension at 72°C for 10 min.	de las Rivas et al. (2006)
106/107	95°C 5 min, 25 cycles of 95°C 45 s, 54°C 1 min, 72°C 1 min with a final extension at 72°C for 10 min.	de las Rivas et al. (2005)

### BA Production From Different Amino Acid Media

Phenotypic analysis of the 27 strains was conducted by adding different substrates. The isolates were cultivated in differential amino acid decarboxylase media as described in the Detection of Amine-Forming Ability section, at 37°C for 48 h. BA analysis from bacterial cultures was performed by RP-HPLC.

### BA Production From Different pH Values

The effect of acid stress on bacterial amine-forming ability was analyzed. Isolates were cultivated in mix-amino acid decarboxylase media as described in “Detection of Amine-Forming Ability” section, at 37°C for 48 h. The pH of the media was adjusted to a range of values (pH 4.50, 5.00, 6.00, and 6.70) by adding disodium hydrogen phosphate–citric acid. BA analysis from bacterial cultures was performed by RP-HPLC.

### Determination of pH and Cell Turbidity

Isolates were cultivated in mix-amino acid decarboxylase media as described in “Detection of Amine-Forming Ability” section. The isolates were grown at 37°C for 0, 4, 8, 12, 24, 36, and 48 h. Then, the 600-nm absorbance and pH of the solution were determined with a spectrophotometer (721G, INESA) and a pH meter (PHS-3E, INESA), respectively.

### Statistical Analysis

Data were processed by analysis of generalized linear model. All statistical procedures were computed using SPSS 17.0.

## Results and Discussion

### Isolation of Strains and Determination of Potential BA Production

A total of 97 isolates were collected from Suan yu. Table 4 shows the BA content and distribution of types in 97 strains of enteric bacteria. The table shows that 97 strains were divided into four grades according to their ability to produce amines. Enteric bacteria produced particularly high amounts of putrescine, followed by histamine and cadaverine. About 33.0% (32/97) of the strains produced more than 900 mg/L of putrescine. The percentage of putrescine content between 500 and 900 mg/L was 48.5% (47/97). Meanwhile, 2.1% (2/97) of the strains produced more than 900 mg/L of histamine. The percentage of histamine content between 500 and 900 mg/L was 5.2% (5/97). The content of histamine was between 100 and 500 mg/L, accounting for 41.2% (40/97). However, no strain produced cadaverine content higher than 500 mg/L. The percentage of cadaverine content between 100 and 500 mg/L was 16.5% (16/97), and 83.5% (81/97) of the strains were below 100 mg/L. As expected, the enteric bacteria detected were dominantly putrescine, histamine, and cadaverine producers (Bjornsdottir et al., 2009; Torido et al., 2012). Similarly, Kim et al. (2009) found that 23 strains of *Enterobacter aerogenes* produce large numbers of putrescine, histamine, and cadaverine, and 33 strains of two *Enterobacter* spp. produce abundant putrescine and cadaverine but less histamine. In this work, none of the tested strains produced tyramine under the present assay conditions. Our obtained results agree with previous studies by de las Rivas et al. (2006), Ladero et al. (2012), and Tittarelli et al. (2019). The results described above indicate that the types and abilities of BAs produced by strains of enteric bacteria in Suan yu were different. The reasons for these differences are species and strain specific, diversity of amino acid decarboxylase genes, and effects of environmental factors (ripening temperature, pH, NaCl, oxygen, Aw). In particular, pH is one of the most important environmental factors (Coton et al., 2010; Chiara et al., 2014; Ozogul et al., 2015).

**TABLE 4:** The BAs content and distribution of types in 97 strains of enteric bacteria.

**BAs content (mg/L)**	**Histamine**	**Putrescine**	**Cadaverine**
≥900	2	32	0
500 ≤ x < 900	5	47	0
100 ≤ x < 500	40	9	16
x < 100	50	9	81
*N*	97	97	97

*x, biogenic amines content; N, numbers.*

### DNA Extraction and Strain Identification

Twenty-seven strains of high-yield putrescine, histamine, and cadaverine were selected from 97 strains for follow-up study. The 16S rDNA of 27 strains was amplified with the universal primers 27F/1492R, and these sequences were analyzed with the BLAST (NCBI). The results are listed in Table 5. Strains were assigned to the different species: two strains of *Enterobacter asburiae*, two strains of *Klebsiella aerogenes*, three strains of *Klebsiella oxytoca*, one strain of *Klebsiella pneumoniae*, five strains of *Enterobacter hormaechei*, three strains of *Enterobacter ludwigii*, seven strains of *Morganella morganii*, one strain of *Citrobacter youngae*, two strains of *Enterobacter cloacae*, and one strain of *Enterobacter* sp.

**TABLE 5:** Identified bacteria of twenty-seven strains of high-yield BAs isolated from Suan yu by 16S rDNA.

**Strain number**	**Nucleotide sequence identification details**		
	**Closest relative**	**NCBI accession number**	**Sequence homology (%)**
26C3	*Enterobacter asburiae*	LC415612.1	100.00
41C3	*Enterobacter asburiae*	MK459533.1	99.93
10C2	*Klebsiella aerogenes*	LR607333.1	100
13C2	*Klebsiella aerogenes*	LR607333.1	99.93
3C3	*Klebsiella oxytoca*	KY614353.1	99.93
7C3	*Klebsiella oxytoca*	KY614353.1	99.93
9C3	*Klebsiella oxytoca*	KF224907.1	99.86
47C2	*Klebsiella pneumoniae*	MT102629.1	100.00
17C2	*Enterobacter hormaechei*	MK286959.1	100.00
17C3	*Enterobacter hormaechei*	MK286959.1	100.00
20C2	*Enterobacter hormaechei*	MK286959.1	100.00
23C3	*Enterobacter hormaechei*	MK286959.1	99.71
29C2	*Enterobacter hormaechei*	MK286959.1	100.00
26C1	*Enterobacter ludwigii*	KF668468.1	100.00
30C1	*Enterobacter ludwigii*	KF668468.1	100.00
33C1	*Enterobacter ludwigii*	KF668468.1	100.00
1C1	*Morganella morganii*	MF033453.1	99.86
6C1	*Morganella morganii*	KC595561.1	100.00
11C1	*Morganella morganii*	KC595561.1	100.00
35C3	*Morganella morganii*	MF033453.1	99.93
42C2	*Morganella morganii*	KC595561.1	99.93
45C2	*Morganella morganii*	MF033453.1	99.93
45C3	*Morganella morganii*	MF033453.1	99.85
33C2	*Citrobacter youngae*	MG428756.1	99.93
29C3	*Enterobacter cloacae*	LT978460.1	100
32C3	*Enterobacter cloacae*	LT978460.1	100
29C1	*Enterobacter* sp.	KU324466.2	98.71

### Genotypic Diversity of BA Production by Enteric Bacteria

**FIGURE 1:**
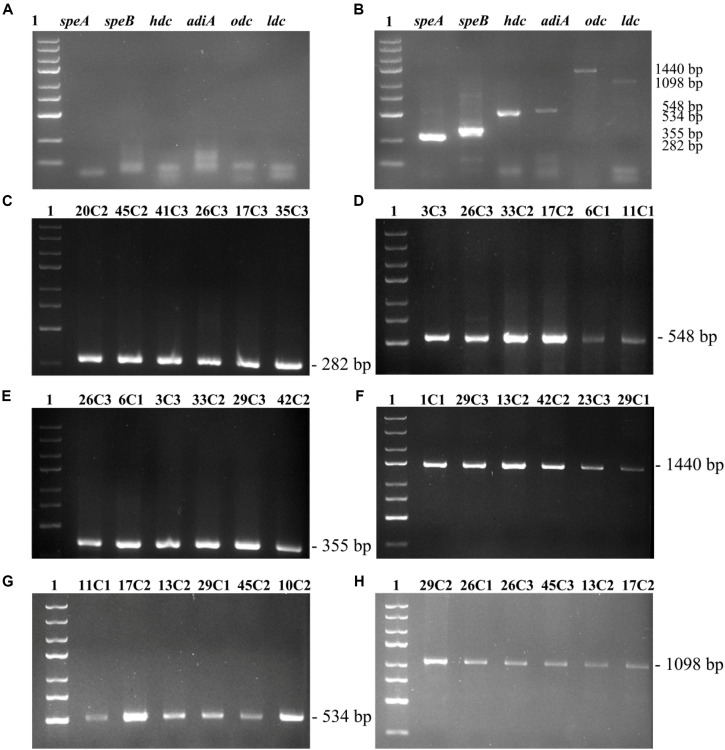
To detect the *speA*, *adiA*, *speB*, *odc*, *hdc*, *ldc* genes by PCR. **(A)** Negative control. **(B)** Positive control. **(C)** *speA* gene fragment. **(D)** *adiA* gene fragment. **(E)** *speB* gene fragment. **(F)** *odc* gene fragment. **(G)** *hdc* gene fragment. **(H)** *ldc* gene fragment. Lane 1, 5000 bp DNA ladder; lanes 2–7, PCR products by DNA from part of 27 strains.

The primer pair PUT1-F/PUT1-R was used to detect the *odc* gene; all the 27 strains produced amplicons of the expected sized 1,440 bp (Figure 1F). Similar results were previously observed by de las Rivas et al. (2006), who found that some strains (*E. coli* MG 1655, *M. morganii* CECT 173T, *Lactobacillus* sp. 30a, *Serratia liquefaciens* IFI-65, and *Oenococcus oeni* RM83) contain the *odc* gene. Moreover, the presence of the corresponding *speA* and *adiA* genes was demonstrated by PCR assay with adc5F/adc5R and adiA3F/adiA3R primers, which amplified fragments of 282 and 548 bp, respectively (Figures 1C,D). A 355-bp partial *speB* gene fragment was amplified with the primer set agm4F/agm4R to detect the agmatinase gene (Figure 1E). In this work, all tested strains carried *speA*, *speB*, and *adiA* genes. The obtained results were in agreement with the findings of Wunderlichová et al. (2013), who described primers adc5F/adc5R, adiA3F/adiA3R, and agm4F/agm4R that amplify corresponding fragments of the *speA*, *adiA*, and *speB* genes in a high number of putrescine-producing strains.

The primer pair 106/107 was used to detect the *hdc* gene. Thirteen strains produced amplicons of the expected size of 534 bp (Figure 1G). Our obtained results agree with the previous study by de las Rivas et al. (2005) who amplified a 534-bp fragment of the *hdc* gene in *K. planticola* CECT 843 and *M. morganii* CECT 173. Moreover, the primer couple CAD1-F/CAD1-R was used to detect the *ldc* gene wherein PCR products were 1,098 bp. In this work, all tested strains carried the *ldc* gene (Figure 1H). Similar results were previously observed by de las Rivas et al. (2006), who detected the *ldc* gene of Gram-negative bacteria by CAD1-F/CAD1-R. Curiel et al. (2011) investigated the BA genes of *Enterobacteria* isolated from fresh sausage packages, indicating that a large number of *Enterobacteria* species are cadaverine-producer strains possessing the ldc gene. The PCR reaction containing all six primer pairs was validated on known BA-producing represented by three strains (Section 2.2). PCR negative control without DNA (Figure 1A). PCR products by DNA from *Photobacterium phosphoreum* CECT 4192 (*hdc*), *S. sonnei* CECT 457 (*odc*), *Escherichia coli* K-12 substr. MG1655 (*speA*, *speB*, *adiA*) (Figure 1B, positive control).

### Phenotypic Analysis for BA Production by Enteric Bacteria

**FIGURE 2:**
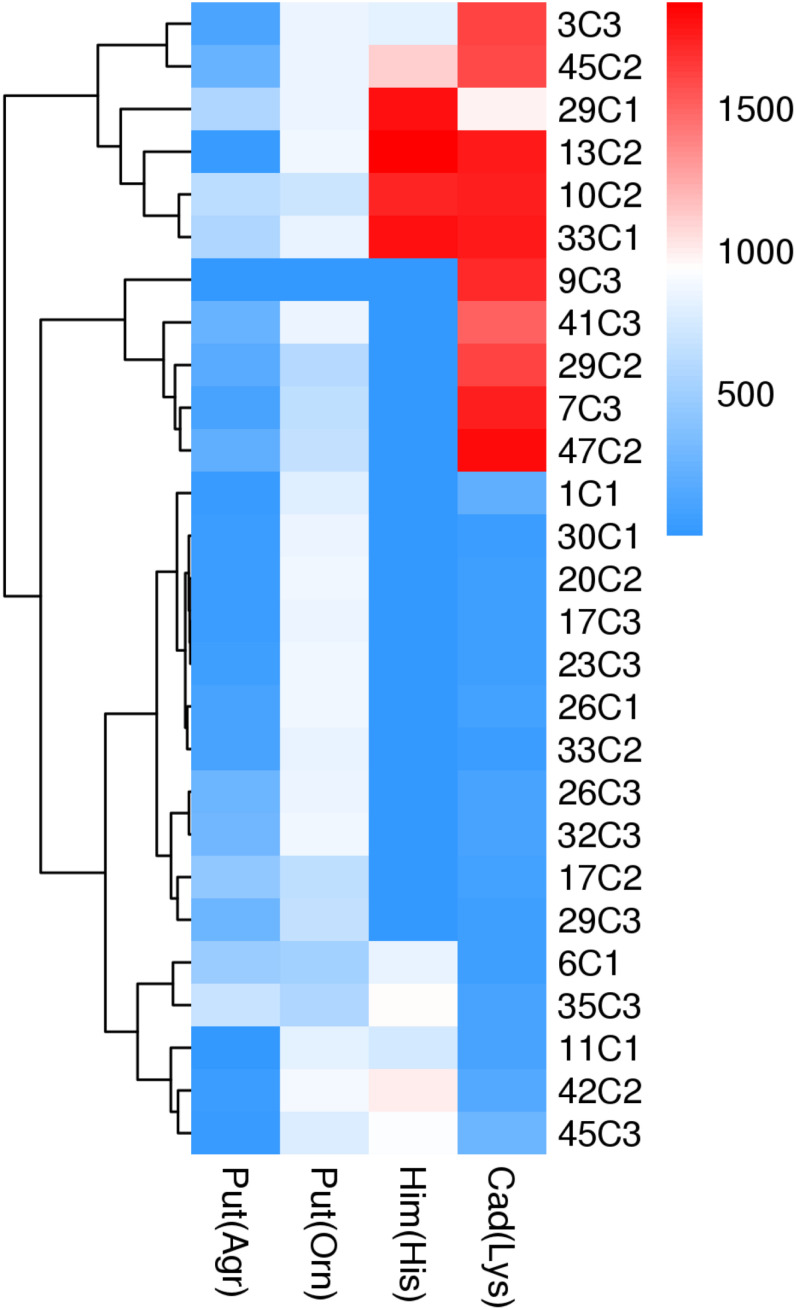
BA production from different amino acid as substrate by enteric bacteria. BA: histamine (Him), putrescine (Put), cadaverine (Cad). Arginine (Arg), ornithine (Om), histidine (His), lysine (Lys).

Phenotypic analysis of the 27 strains was conducted by adding different substrates, and the correlation between the genotypic and phenotypic data was explored. As shown in Figure 2, BAs were formed by the decarboxylation of the corresponding amino acids and amino acid precursors, and the phenotypic and genotypic data coincided. The results show that the phenotypic and genotypic data coincided.

The 27 tested strains were putrescine positive as determined by RP-HPLC in medium supplemented with L-ornithine hydrochloride or L-arginine. In the two metabolic pathways of putrescine production, the phenotypic and genotypic data coincided in 27 strains tested. *M. morganii* strain 42C2 produced the highest content of putrescine in the medium supplemented with L-ornithine hydrochloride (880 mg/L), followed by *E. hormaechei* 20C2 (873 mg/L), and then *K. aerogenes* 13C2 (871 mg/L). *M. morganii* 35C3 produced the highest content of putrescine in the medium supplemented with L-arginine (681 mg/L).

Thirteen of the 27 tested strains were histamine positive as determined by phenotypic analysis. In addition, 37.0% (10/27) of the strains produced more than 800 mg/L of histamine. The highest histamine formation was shown by *K. aerogenes* 13C2 (1,869 mg/L), followed by *Enterobacter* sp. 29C1 (1,812 mg/L), and then *E. ludwigii* strain 33C1 (1,803 mg/L). The 27 tested strains were cadaverine positive as determined by RP-HPLC in medium supplemented with L-lysine, and the phenotypic and genotypic data coincided. *K. pneumoniae* 47C2 produced the largest amount of cadaverine (1,821 mg/L), followed by *E. ludwigii* 33C1 (1,765 mg/L), and then *K. aerogenes* 13C2 (1,771 mg/L). The cadaverine content of the other strains ranged from 50 to 276 mg/L.

Notably, *K. aerogenes* 13C2 produced particularly high amounts of putrescine, histamine, and cadaverine. In addition, limited information exists regarding the formation of BAs by *K. aerogenes* isolated from fish and fish products.

### Effects of Different pH Values on the Biogenic Amine Contents and Types

#### Effects of Different pH Values on the Total BA Content of the 27 Strains

**FIGURE 3:**
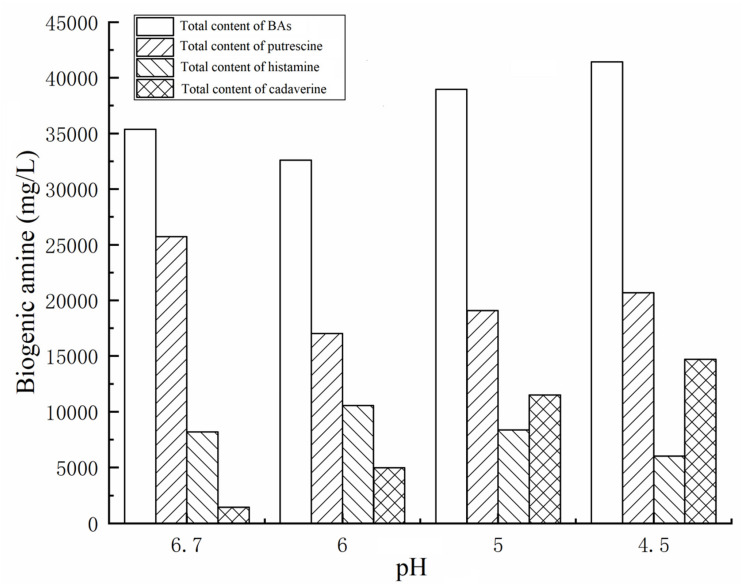
BAs contents of twenty-seven strains of enteric bacteria at different pH values.

In the present work, to understand the effect of different pH values on BA production by the 27 strains under mixed substrates, we calculated the total BA content of the 27 strains. As the pH decreased from 6.70 to 4.50, the total content of BAs of the 27 strains reached the maximum at pH 4.50 and the lowest at pH 6 (Figure 3). However, the content of BAs decreased sharply at pH 4 because high acid inhibited the growth of enteric bacteria, thus inhibiting the production of BAs (data not shown).

#### Effects of Different pH Values on the Amines Produced by 27 Strains

**FIGURE 4:**
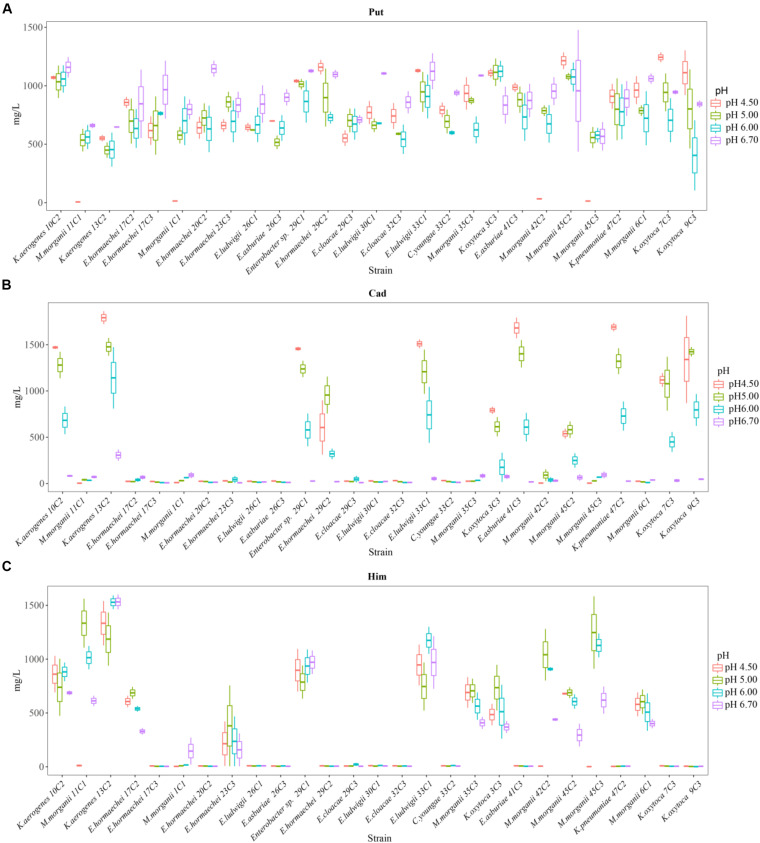
Putrescine content **(A)**, histamine content **(B)**, and cadaverine content **(C)** produced by different strains of enteric bacteria under the stresses of different pH values.

We also calculated the total contents of putrescine, histamine, and cadaverine, respectively (Figure 3), showing the amines produced by each strain (Figures 4A–C). As shown in Figures 3, 4A–C, the different pH levels affected the BA-producing abilities of the 27 strains in a strain-dependent rather than species-specific manner. These results were in agreement with the findings of Coton et al. (2010) and Ozogul et al. (2015). The cooperation of various amino acid decarboxylase pathways under acid stress and the effect of pH on the activity of amino acid decarboxylase (Table 6 and Figures 2–4, 6).

**TABLE 6:** BAs formed by different strains of the same species in single substrate and mixed substrate.

**Strain number**	**Strains**	**BA**	**Biogenic amines content (mg/L)**	
			**pH 6.7 (Single)**	**pH 6.7 (Mix)**
13C2	*Klebsiella aerogenes*	Put	900	652
		Cad	1771	246
		Him	1869	1601
10C2	*Klebsiella aerogenes*	Put	1339	1071
		Cad	1751	94.6
		Him	1723	708
35C3	*Morganella morganii*	Put	1254	1099
		Cad	104	109
		Him	941	4648
45C2	*Morganella morganii*	Put	1100	1478
		Cad	1596	100
		Him	1113	400

*Him, histamine; Put, putrescine; Cad, cadaverine. Single: single substrate. Mix: mix substrates.*

##### Effect of different pH levels on putrescine content

As shown in Figures 3, 4A, pH considerably affected the putrescine-producing ability of the tested bacteria (*P* < 0.01). As the pH decreased from 6.70 to 4.50, the total amount of putrescine of the 27 strains reached the maximum at pH 6.7 and the lowest at pH 6.00 (Figure 3). When the pH dropped to 5.00 and 4.50, the total content of putrescine increased but did not exceed the total content of putrescine at pH 6.70. This result indicates that the optimum pH for putrescine production is 6.70 (Figures 2, 3, 4A). The putrescine production of *M. morganii* 42C2, 11C1, 1C1, and 45C3 were ND at pH 4.50 because the high acidity inhibited their growth. The highest putrescine content was produced by *M. morganii* 45C2 (1,478 mg/L) at pH 6.7, followed by *E. hormaechei* 20C2 (1,215 mg/L) (Figure 4A).

The content of putrescine was the highest at pH 6.70 (Figure 4A). At this pH, the combination of *adiA* and *odc* consumed arginine and ornithine, respectively, in the environment to produce a large amount of putrescine, and the contribution of ornithine decarboxylase (*odc*) to the production of putrescine was greater than that of arginine decarboxylase *adiA*. The following results illustrate the above inferences. At pH 6.70, the total amount of putrescine obtained by adding two types of single substrates was basically the same as that obtained by mixing substrates (Figures 2, 4A). For putrescine production under single substrate (Figure 2), when the pH was 6.70, the amount of putrescine produced by adding L-ornithine hydrochloride as the substrate was greater than that of L-arginine as the substrate. Usheer et al. (2011) have reported that the optimum pH for *odc* is 7.00. In addition, *adiA* alone has a peak activity at pH 5.00. The activity of *adiA* drops off very dramatically at pH values higher than 6.00 and lower than 4.00, and the expression of *adiA* was the highest at pH 4.50 (citric acid buffer) (Álvarez-Ordóñez et al., 2010; Usheer et al., 2011). As shown in Figures 3, 4A, the production of putrescine at pH 5.00 and 4.50 was higher than that at pH 6.00. Our results also demonstrate that the putrescine produced by adding arginine at pH 4.50 (data not shown) was higher than that at pH 6.70 (Figure 2), and the putrescine produced by adding L-ornithine hydrochloride at pH 4.50 (data not shown) was slightly lower than that at pH 6.70. The pH seems not to affect *odc* expression levels but the activity of *odc* enzymes decreases under acidic conditions (de las Rivas et al., 2008; Usheer et al., 2011). However, at pH 4.50, the total amount of putrescine obtained by adding two types of single substrates was higher than that obtained by mixing substrates (data not shown). The reason might be that other metabolic pathways (*hdc*, *ldc*) are also involved in the acid stress response, thus, reducing the formation of putrescine. At pH 4.50, which of the two pathways contributes the most in the production of putrescine in the mixed substrates? We need to do in-depth research.

##### Effect of different pH levels on cadaverine content

As shown in Figures 3, 4B, pH significantly affected the cadaverine-producing ability of the tested bacteria (*P* < 0.01). As the pH was decreased from 6.70 to 4.50, the total cadaverine content increased gradually (Figure 4B). The total cadaverine content of the 27 strains reached the maximum at pH 4.50 and the lowest at pH 6.70 (Figure 3). At pH 4.50, 25.9% (7/27) of the strains produced more than 1,400 mg/L of cadaverine. The highest cadaverine content was produced by *K. aerogenes* 13C2 (1,862 mg/L), followed by *K. oxytoca* 9C3 (1,813 mg/L), *E. asburiae* 41C3 (1,793 mg/L), *K. pneumoniae* 47C2 (1,736 mg/L), *Enterobacter* sp. 29C1 (1,477 mg/L), and *K. aerogenes* 10C2 (1,453 mg/L) (Figure 4B). The obtained results were in agreement with the findings of a previous study possibly because abundant *ldc* expression was induced under acid stress (Bover-Cid et al., 2001; Greif et al., 2006). Some studies have indicated that the *ldc* expression of *E. coli* is induced at acidic pH as well as by high concentrations of the respective amino acids (Park et al., 2017). The optimum pH level for the decarboxylation activity of *Klebsiella* and *E. coli* is 5.50 (Li et al., 2014). In addition, Tanaka et al. (2008) indicated that the addition of lysine improves *Vibrio parahaemolyticus* acid survival, and *ldc* expression at pH 5.00 is higher than that at pH 5.50, suggesting that *ldc* plays a role in the adaptive acid tolerance response. Our findings showed that the optimum pH for the *ldc* activity of enteric bacteria was 4.50–5.00 in the medium with mixed amino acid (Figures 3, 4B). Of the strains produced below 33 mg/L of cadaverine at pH 4.50, 59.3% (16/27) showed higher production of putrescine or histamine, especially putrescine (Figure 4).

##### Effect of different pH levels on histamine content

As shown in Figures 3, 4C, pH had a significant effect on the histamine-producing ability of the tested bacteria (*P* < 0.05). As the pH decreased from 6.70 to 4.50, the total histamine amount of the 27 strains reached the maximum at pH 6.00 and the lowest at pH 4.50 (Figure 3). The total amount was similar at pH 6.70 and 5.00 (Figure 4C). At pH 6.00, 40.7% (11/27) of the strains produced more than 500 mg/L of histamine, and six strains produced more than 900 mg/L of histamine. High-level histamine was produced by *K. aerogenes* 13C2 (1,465 mg/L), *K. aerogenes* 10C2 (969 mg/L), *Enterobacter* sp. 29C1 (1,090 mg/L), *E. ludwigii* 33C1 (1,049 mg/L), *M. morganii* 45C3 (1,239 mg/L), and *M. morganii* 11C1 (905 mg/L) (Figure 4C).

Greif et al. (2006) reported that high amounts of histamine are produced at pH 5.80–6.60 (309–328 μg/cm^3^). Similarly, the optimum pH for the *hdc* activity of some bacteria, such as *Photobacterium damselae*, *M. morganii*, *Raoultella planticola*, *E. aerogenes*, and *Proteus vulgaris* ranged from 5.00 to 6.50 (Bjornsdottir-Butler et al., 2015). In addition, previous qRT-PCR experiments showed that the *hdc* and *hisRS* genes are highly induced under acidic and histidine-rich conditions, and these results support the hypothesis that the *hdc* pathway in histidine producers plays a role in acid survival (Chiara et al., 2014). According to the above literature, the suitable pH for histamine production is between 5.00 and 6.60. Our findings showed that the optimum pH for the *hdc* activity of enteric bacteria was 5.00–6.00 in the medium with mixed amino acid (Figures 3, 4C). The obtained results were in agreement with a previous study (Bjornsdottir-Butler et al., 2015; Hu et al., 2015).

##### Mutual cooperation of different amine production pathways on different pH values

Interestingly, the content of BAs produced by the same strain under a single amino acid and mixed amino acid enrichment medium was different (Table 6 and Figures 2, 4). For instance, at pH 6.70, some strains produced particularly high amounts of putrescine, histamine, and cadaverine in a single amino acid enrichment medium, but they produced large amounts of putrescine and less histamine and cadaverine in the mixed amino acid enrichment medium. As the pH was decreased from 6.70 to 4.50, the contents of histamine and cadaverine increased, especially cadaverine (Figure 4). Histamine production was the highest when putrescine production was the lowest at pH 6.00 (Figure 3). The total cadaverine content of the 27 strains reached the maximum at pH 4.50 and the lowest at pH 6.70 (Figures 3, 4B). At pH 4.50, the total amount of putrescine obtained by adding two types of single substrates was higher than that obtained by mixing substrates (data not shown). These results indicated that pH can affect the type and ability of BAs formed by enteric bacteria from Suan yu. This phenomenon may be attributed to the cooperation of various amino acid decarboxylase pathways under acid stress and to the effect of pH on the activity of amino acid decarboxylase (Table 6 and Figures 2–4).

**FIGURE 5:**
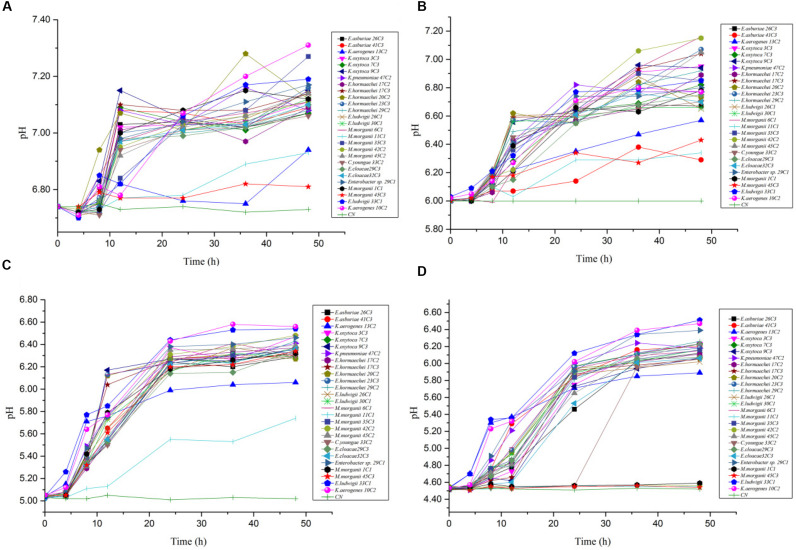
The pH value change rules of different strains of enteric bacteria under the stresses of different pH values. **(A)** pH 6.70. **(B)** pH 6.00. **(C)** pH 5.00. **(D)** pH 4.50.

**FIGURE 6:**
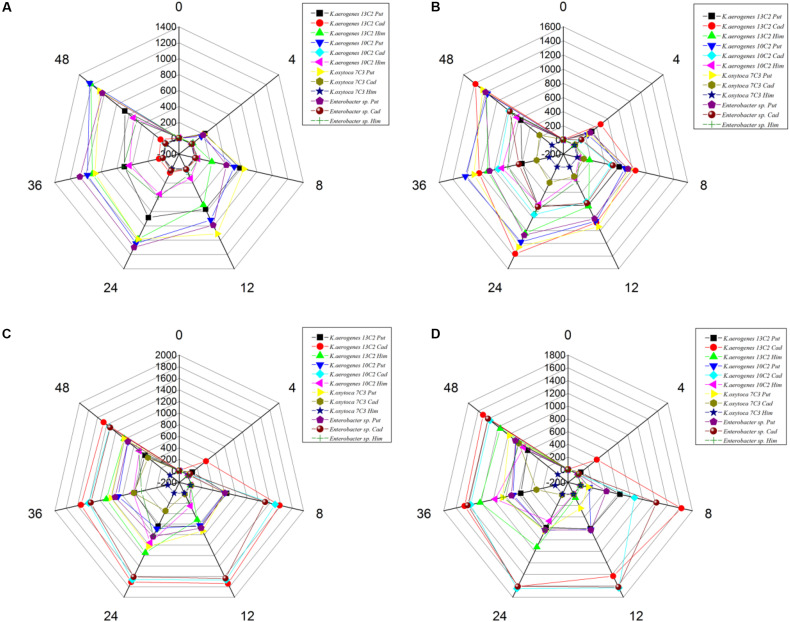
The types and content of BAs by different strains of enteric bacteria for 0, 4, 8, 12, 24, 36, 48 h under the stresses of different pH values. **(A)** pH 6.70. **(B)** pH 6.00. **(C)** pH 5.00. **(D)** pH 4.50.

**FIGURE 7:**
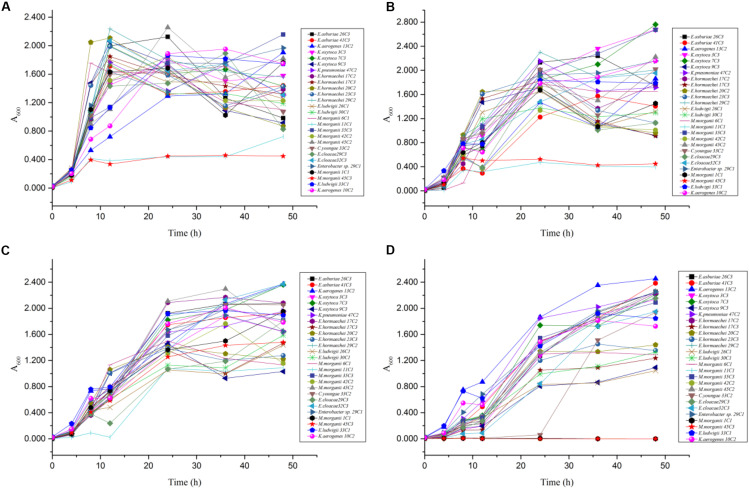
The OD_600_ value change rules of different strains of enteric bacteria under the stresses of different pH values. **(A)** pH 6.70. **(B)** pH 6.00. **(C)** pH 5.00. **(D)** pH 4.50.

In the mixed amino acid enrichment medium, excessive production of BAs is unnecessary to balance intracellular and extracellular pH at pH 6.70. As the cultivation time was prolonged from 0 to 48 h, the pH value of the cultures reached the peak of growth in 12 h (Figure 5A). Subsequently, the pH evolution of the cultures stabilized. The pH value of the medium changed from 6.70 to 7.31, with a span of 0.10–0.61. The content of BAs in the culture process of strains *K. aerogenes* 10C2, 13C2, and *Enterobacter sp.* 29C1 at this pH was measured. A large amount of putrescine was produced first after 4 h of cultivation, and the histamine and cadaverine contents were lower than 10 mg/L. The histamine content increased rapidly after 8 h of cultivation. The content of cadaverine did not change considerably in 48 h (Figure 6A). The results showed that the production of putrescine and histamine raised the pH of the broth. The A_600_ of cell turbidity in the broths with amino acids was measured. At 8 h, the OD600 value reached above 0.6–0.8. At 12 h, it reached the maximum value (except 45C3 and 11C1). Subsequently, it decreased with prolonged culture time but remained above 0.8 (Figure 7A). This result indicated that the growth of the strain was normal. However, as the pH was decreased from 6.7 to 4.5, more BAs need to be produced to balance the intracellular and extracellular pH, thus increasing the decarboxylation of other amino acids. As shown in Figures 5B–D, the pH of the medium under acid stress increased rapidly after 4 h. Meanwhile, the pH of the broth at pH 6.00, 5.00, and 4.50 reached a stable level at 36, 24, and 36 h, respectively. The change spans of the pH value were 0.29–1.16, 0.74–1.56, and 0.04–2.01, respectively (Figures 5B–D). Figure 6B shows that the contents of putrescine and cadaverine increased for 4 h, and the content of histamine increased gradually after 4 h in the pH 6.00 medium. In the pH 5.00 and 4.50 media (Figures 6C,D), a large amount of cadaverine was produced after 4 h of cultivation, followed by putrescine. After 12 h of cultivation, the histamine content increased rapidly. The pH 5.00 medium can quickly reach pH stability because the pH was the optimal pH for *adiA* and *ldc*. When the optimal pH for *hdc* was reached, the histamine production pathway participated in the pH adjustment. The above results illustrate that different BA production pathways work together to improve the extracellular pH. The OD600 values of the pH 6.00, 5.00, and 4.50 broths reached 0.6–0.8 at 10, 12, and 20 h (Figures 7B–D), respectively, which were delayed for 2, 4, and 12 h, respectively, compared with that of the pH 6.7 broth (Figure 7A). In addition, the OD600 values reached the growth peak at 24, 36, and 48 h, respectively, which were delayed for 12, 24, and 36 h (Figures 7B–D), respectively, compared with pH 6.7 (Figure 7A). As shown in Figures 3, 6, 7, acid stress caused the growth delay, which did not affect the production of putrescine, cadaverine, and histamine contents; on the contrary, acid stress can increase the contents of these BAs. *K. oxytoca* 7C3 does not carry the *hdc* gene and does not produce histamine. A large amount of putrescine was produced from pH 6.70–4.50, and part of cadaverine was used to adjust the acid environment. Nevertheless, it produced particularly high amounts of cadaverine in a single substrate.

The above-mentioned results indicated that pH can considerably affect the BA-producing ability of the tested bacteria. *odc*, *adiA*, *speB*, *ldc*, and *hdc* were important for enteric bacteria survival under acidic conditions, and these pathways should be collaborative. At pH 6.70 and 6.00, the putrescine, histamine, and cadaverine production dominated in the regulation of extracellular acid environment in most strains of enteric bacteria. At pH 5.00 and 4.50, the putrescine and cadaverine production in the regulation of extracellular acid environment in most strains of enteric bacteria and the putrescine and histamine production are the main pathways in a few enteric bacteria. Decarboxylase was strain specific rather than species specific (*P* < 0.01) (Table 6 and Figures 2–4, 6).

## Conclusion

Out of 97 strains showing high BA levels, 88, 47, and 16 strains produced more than 100 mg/L of putrescine, histamine, and cadaverine, respectively. All tested strains carried the corresponding amino acid decarboxylase genes of putrescine (*odc*, *speA*, *speB*, and *adiA*) and cadaverine (*ldc*). In addition, 13 of 27 strains carried histidine decarboxylase gene (*hdc*). *odc*, *adiA*, *speB*, *ldc*, and *hdc* were important for enteric bacteria survival under acidic conditions, and these pathways should be collaborative. Decarboxylase was strain specific rather than species specific (*P* < 0.01). Our results indicated that acid stress caused the growth delay but can increase the contents of putrescine, histamine, and cadaverine. On the one hand, this study can provide a reference for the genotypic diversity of decarboxylase of BA-producing bacteria and the effect of pH on the types and abilities of BAs produced by enteric bacteria in protein-rich fermented fish. On the other hand, fermented low-acid and high-protein foods must strictly prevent intestinal bacterial contamination; otherwise, large amounts of BAs will be generated. Unqualified products will cause economic loss and safety hazards.

## Data Availability Statement

The raw data supporting the conclusions of this article will be made available by the authors, without undue reservation, to any qualified researcher.

## Ethics Statement

Ethical review and approval was not required for the animal study because Suan yu is a local snack that has been consumed for a long time by ethnic minority people in southwest China.

## Author Contributions

QY wrote the main manuscript text. JM and LL prepared Figures 1–3 and Tables 1–6. WZ prepared Figures 5–7 and modified the manuscript. LH, LD, and CY provided advice for the manuscript. XZ directed and modified the manuscript.

## Conflict of Interest

The authors declare that the research was conducted in the absence of any commercial or financial relationships that could be construed as a potential conflict of interest.
